# *In vitro *effect of fluoride oral hygiene tablets on artificial caries lesion formation and remineralization in human enamel

**DOI:** 10.1186/1472-6831-9-25

**Published:** 2009-10-02

**Authors:** Peter Gängler, Thomas Kremniczky, Wolfgang H Arnold

**Affiliations:** 1 Faculty of Dental Medicine, University of Witten/Herdecke, Witten, Germany

## Abstract

**Background:**

Aim of this *in-vitro*-study was to assess the remineralization potential of a tooth cleaning tablet with different fluoride content.

**Methods:**

Twenty three caries free impacted third molars were examined, enamel surfaces were wax coated leaving two 3 × 4 mm windows for exposure to demineralization/remineralization cycles. The teeth were randomly assigned to 4 groups of 5 control and 6 experimental teeth. Demineralization by standardised HEC-gel, pH 4.7 at 37°C for 72 h, was alternated by rinsing in remineralization solution, pH 7.0 at 37°C for 72 h, total challenge time 432 h. The negative control group N was treated during remineralization cycles with saline; positive control group P was treated with remineralization solution; experimental group D1 was exposed to remineralization solution containing Denttabs^®^-tablets with 1450 ppm F; experimental group D2 was exposed to remineralization solution and Denttabs^®^-tablets with 4350 ppm F. Each tooth was cut into serial sections and analyzed by polarized light microscopy for assessment of the different zones of white-spot lesions in 3 representative sections. Statistical analysis was based on the *Mann-Whitney*-Test.

**Results:**

Both control groups N(-) and P(+) exhibited characteristic white-spot lesions. The remineralization and the demineralization inhibition of the lesions increased considerably from N<P < D1<D2. Denttabs^®^-2 administration showed partial/total remineralization including lamination and/or disappearance of the body of the lesion. The different results of all 4 groups were statistically highly significant (p < 0.01) with both tests.

**Conclusion:**

Based on these results the novel Denttabs^® ^formulation represents a highly effective oral hygiene product and the remineralization is correlated to the fluoride content.

## Background

Fluoride dentifrices are widely used, and it is experimentally well established that they contribute to remineralization of incipient caries lesions in human and bovine enamel [1-3]. In the last decade many questions have been raised on the bioavailability of fluoride in oral fluids, and new formulations with different fluoride content are under investigation. Recently it has been demonstrated that elevated fluoride products enhance remineralization of advanced bovine enamel lesions [4].

On the other hand, the long or even life long presence of the human dentition combined with dietary influences and individual tooth brushing habits may lead to the risk of erosive and/or abrasive lesions of enamel and especially of root dentin. It was therefore the aim of the development of an oral hygiene agent in tablet form to increase the fluoride availability in oral fluids and to substitute the abrading effect of many traditional toothpastes by a polishing action due to microcrystalline hydroxyethyl cellulose. The resulting product (Denttabs^®^, Prodentum, Berlin, Germany) is rapidly dissolved in saliva, and the fluoride bioavailability immediatedly after tooth brushing and 10 minutes after tooth brushing is higher (Median 165.2 ppm F^-^) compared to a conventional dentifrice foam/saliva mixture (Median 123.7 ppm F^-^) (Naumova et al., Fluoride bioavailability in saliva after using DENTTABS^® ^compared to dentifrice, submitted).

The oral hygiene tablets have also been clinically tested, and the plaque reducing efficiency was similar to a conventional dentifrice in a field study as well as in a controlled clinical trial [5].

The question remained whether an additional fluoride enhancement of remineralization could be expected for an elevated fluoride content of 4350 ppm versus 1450 ppm in Denttabs^®^. The higher fluoride concentration in 0.33 g tablets is equal to 1450 ppm F^- ^per 1.00 g toothpaste as the common amount per application.

It was therefore the aim of this study to assess the relative efficiency of two fluoride treatments on demineralization and remineralization of human dental enamel in a pH cycling model. Qualitative and semi-quantitative polarized light microscopy of serial ground sections was used to detect the morphology of lesions and to assess the pore volume of de- and remineralization. Polarized light microscopy has been chosen as the method to determine the morphology of the different lesions because it allows distinguishing between the different lesion zones. Also the extent of the different lesions zones could be measured exactly.

## Methods

### Tissue

Twenty three completely impacted human third molars were used in agreement with GLP instructions governing the use of human tissue. These teeth were selected because of the uniform enamel structure with no individual challenges due to the non-exposure to the oral cavity. Immediately after clinically indicated surgical removal they were thoroughly cleaned of organic debris and stored in saline containing 0.1% thymol. No further surface treatment of the enamel was applied. The teeth were than coated with wax leaving a 3 × 4 mm window on the buccal and lingual smooth surfaces and finally randomly assigned to 4 groups (Tab. 1).

**Table 1:** Overall experimental design

**Group**	**Demineralisation**	**Remineralization**	**Teeth**	**Lesions**
**N** (negative control)	Standardized HEC-gel pH 4.7, 37°C	Saline 37°C	5	10
		
**P** (positive control)		Remin. solution pH 7.0, 37°C	6	12
		
**D1** (experimental)		Remin. solution + Denttabs^® ^**1450 **ppm F- pH 7.0, 37°C	6	12
		
**D2** (experimental)		Remin. solution + Denttabs^® ^**4350 **ppm F- pH 7.0, 37°C	6	12

### Demineralization/Remineralization, pH-Cycling

pH-cycling conditions were chosen to create advanced artificial enamel lesions on natural smooth surfaces. Each cycle was scheduled for 3 days and was repeated 6 times. The experimental period of pH-cycling lasted therefore 18 days. After demineralization and remineralization (except for the negative control group) the specimens were rinsed in distilled water to remove excess treatment gel solution. Demineralization gel contained 1.5 mM CaCl_2_, 0.9 mM KH_2_PO_4_, 150 mM KCl, 0.1 M sodium acetate buffer, 30 mM acetate in hydroxyehtyl cellulose. The pH was adjusted to 4.7 and controlled before and after each 3 day cycle [6].

Remineralization solutions were comprised of 1.5 mM CaCl_2_, 0.9 mM KH_2_PO_4_, and 150 mM KCl at pH 7.0, again controlled before and after each 3 day cycle.

**Figure 1:**
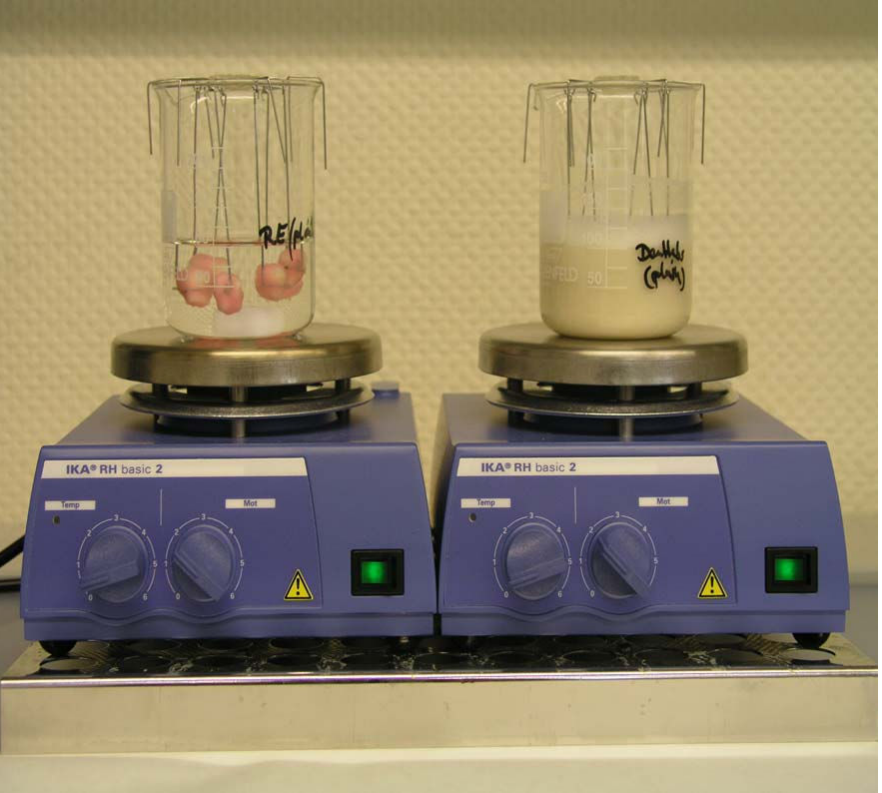
**Setup of the incubation procedure**. The wax coated teeth were fixed to metal wires and were hanging in the different incubation media. The incubation media were constantly slowly agitated with a magnetic stirrer.

The wax coated teeth were fixed with metal wires and hang in the respective solutions (Figure 1) and the volume of each solution was 100 ml. These solutions were constantly agitated using a magnetic stirrer. All cycles were executed under constant climatic conditions at 37°. The window areas were microphotographically controlled before pH-cycling to discard specimens with enamel cracks of iatrogenic scratches and to document the artificial white spot lesions after 18 days.

### Treatment groups

The experimental scheme is presented in Table 1. The negative control group N of 5 teeth with10 lesions underwent demineralization cycles only and was kept during the remineralization cycles in saline. The positive control group P of 6 teeth with 12 lesions was remineralized in the specified solution without fluoride content. The 2 experimental groups (D1 and D2) of 6 teeth with 12 lesions each were exposed to remineralization solution containing Denttabs^® ^with 1450 ppm F^- ^(D1) or Denttabs^® ^with 4350 ppm F^- ^(D2). The oral hygiene tablets contain fluoride from NaF, and the other ingredients according to INCI are microcrystalline hydroxyethyl cellulose, hydrated silica, sodium hydrogen carbonate, sodium laurylsulfate, ascorbic acid, magnesium stearate, aspartame and mint flavor.

The tablets were suspended in the remineralization solution with a ratio of one tablet per 5 ml solution to simulate the maximal bioavailability of fluoride immediately after brushing (Naumova et al., Fluoride bioavailability in saliva after using DENTTABS^® ^compared to dentifrice, submitted).

### Polarized light microscopy (PLM)

After removal of the wax coatings standardized micro photos of all lesions at 10× magnification were taken. Before further processing the roots were removed. All teeth were then dehydrated in graded alcohol and embedded in Technovit 9100 (Kulzer, Weinheim, Germany). Serial ground sections were cut with a saw microtome (LEICA CM 1900, Leica, Wetzlar, Germany) with a thickness of 80 μm in corono-apical direction. Three ground sections per tooth representing two approximal and one middle area of the lesions were used for qualitative and semi quantitative assessment of the lesion morphology.

The qualitative assessment of lesion zone characteristics included the homogeneity of the superficial zone and of the body of the lesion representing the area-specific mineral loss due to changing birefringence of different pore volumes imbibited with Technovit 9100. In addition the presence of laminations in the body of the lesion and the detectable translucent zone was analyzed.

For quantitative analysis the extent of the respective caries-like lesion zones (surface zone, body of the lesion, translucent zone) was measured in μm integrating their maximal and minimal values and continuing in equal distances over the whole lesion until 10 measurements were assessed. Three sections of each lesion were selected for the measurements.

### Statistics

Because the data showed no normal distribution statistical analysis was done using the non parametric Mann-Whitney U test and SPSS 14 as computer program. Significance was determined at p < 0.05.

## Results

### Macroscopic appearance

**Figure 2:**
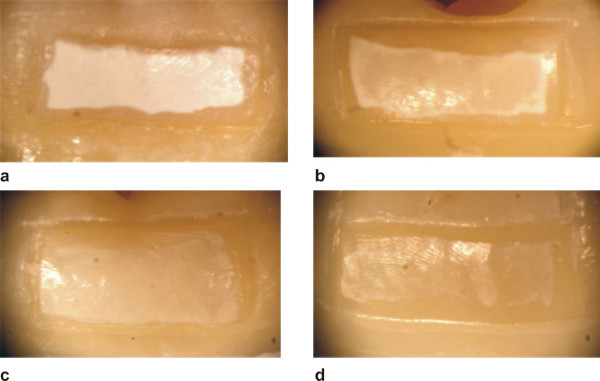
**Appearance of the experimental lesions**. White-spot lesions in the control group N (a), remineralization group P (b), experimental group D1 with Denttabs^® ^1450 ppm fluoride (c) and experimental group D2 with Denttabs^® ^4350 ppm fluoride (d).

In all 4 groups after 18 experimental days artificial caries-like enamel lesions of different intensity were detected. None of these lesions exhibited surface erosion, and the surface morphology at 10× magnification was similar to sound enamel around the lesions. However, there was a marked difference of the appearance of subsurface white-spot lesions. The negative control group N showed rather homogeneous advanced white-spot lesions, whereas in the positive control group P the white spot lesions were less homogeneous with color changes. In contrast both experimental groups D1 and D2 resulted in less intensive white-spot lesions, and the least subsurface demineralization close to non detectable white spots was documented for D2 (Figure 2).

### Qualitative PLM assessment

**Figure 3:**
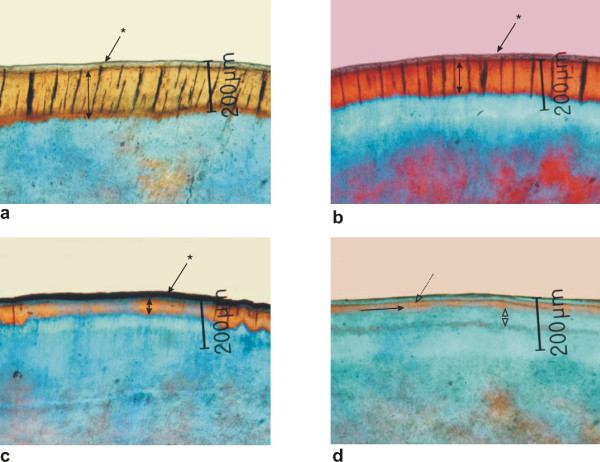
**Polarized light micrographs of morphological examples of the 4 groups of control and experimental teeth**. a) Negative control N: Homogeneous surface zone, demineralized body of the lesion with no laminations; arrow with asterisk = surface zone, double arrow = body of the lesion. b) Positive control P: Homogeneous surface zone, smaller body of the lesion, detectable translucent zone; arrow with asterisk = surface zone, double arrow = body of the lesion. c) Experimental group D1: homogeneous surface zone, small band of the body of the lesion, detectable translucent zone arrow with asterisk = surface zone, double arrow = body of the lesion. d) Experimental group D2: Homogeneous surface zone, disappearing body of the lesion with laminations, broad translucent zone; dotted line with outlined arrow = laminated body of the lesion, arrow = laminations; double outlines arrow = translucent zone.

The characteristic features of 3 ground sections per lesion are summarized in Table 2. The surface zone and the body of the lesion in experimental D1 and D2 groups were less homogenous because of the lower pore volume. Laminations within the body of the lesions were predominantly detected in experimental groups D1 and D2. D2 group showed partial or total remineralization including lamination and/or disappearance of the body of the lesion (Figure 3).

**Table 2:** Qualitative polarized light microscopy with characteristic features of artificial enamel lesions, percentage of serial ground sections

**Group**	**Surface zone homogeneous**	**Body of the lesion homogeneous**	**Presence of laminations**	**Detection of translucent zone**
**N**	100%	90.0%	-	85.0%

**P**	100%	87.5%	8.3%	95.8%

**D1**	83.3%	75.0%	37.0%	100%

**D2**	**70.8%**	**62.5%**	**45.8%**	**100%**

### Quantitative PLM assessment

The results of all measurements of the extent of the surface zone, the body of the lesion, and of the translucent zone are presented in Table 3. The surface zone of group N was significantly more extended compared to all other groups (p < 0.01). The differences in the extent of the body of the lesion were highly significant showing the least demineralization for group D2 with high fluoride content (p < 0.001). The extent of mineral loss per lesion and per group was N>P > D1>D2. The translucent zone was more extended in group D2 and statistically highly different from all other groups (p < 0.001). This was confirmed by the Kruskal - Wallis test (p < 0.001). The results were the same for the overall depth of all lesion zones as well as for the minimal and maximal extent. The most mineral loss was observed in the negative control group N, and the least loss in the fluoride group D2 (N>P > D1>D2).

**Table 3:** Extent of lesion zones in μm in the different groups

	**Surface zone**			**Body of lesion**			**Translucent zone**		
**Group**	**Median**	**Mean**	**STD**	**Median**	**Mean**	**STD**	**Median**	**Mean**	**STD**

**N**	14,2	15,7	5,5	203,5	203,3	40,7	67,8	78,9	30,9

**P**	10,7	10,9	2,9	135,7	132	20,3	89,2	87,6	18,6

**D1**	10,7	10,5	3	71,4	72	13,1	75	79,3	22,6

**D2**	10,7	11,2	5,5	35,7	37,2	13	100	99,4	36,7

## Discussion

Previous studies of the natural history of human enamel caries lesions in deciduous and permanent teeth using three dimensional features of polarized light microscopy have contributed to the understanding of the dynamics of caries progression [7-9]. The same methodology including the 3D-reconstruction of artificial caries-like lesions and the assessment of the volume of the body of the lesion has been exploited for testing various toothpastes [1] and under different pH conditions [10]. The size, volume and configuration of these artificial lesions differ greatly from the typical characteristics of natural early onset, arrested or progressing lesions. However, the lesion zones in polarized light according to Gustafson [11] are the same, and artificially created caries-like lesions are widely used to detect the regulation of mineral deposition and dissolution [3] and to determine the relative efficiency of fluoride toothpastes under pH cycling conditions [2].

The data of the present study confirm the effects of fluoride on demineralization and remineralization of human enamel. It was found that the demineralization regime over 18 days created advanced caries-like lesions with homogeneous surface zones and high pore volumes of the body of the lesion. The extent was around 200 μm and different from the positive control teeth in pH cycling with standard remineralization solution with a depth around 120 μm.

The fluoride availability from 1450 ppm containing oral hygiene tablets in the remineralization solution was equal to 96 ppm (one 0.33 g tablet per 5 ml solution) what corresponds to the fluoride bioavailability from a conventional 1400 ppm F^- ^dentifrice in saliva immediately after tooth brushing (Naumova et al., Fluoride bioavailability in saliva after using DENTTABS^® ^compared to dentifrice, submitted). The elevated fluoride availability from the 4350 ppm containing oral hygiene tablets was 287 ppm what again simulates the bioavailability in saliva after tooth brushing with Denttabs^®^.

In both experimental groups of teeth the mineral loss decreased tremendously and the different extent of the body of the lesion was highly significant. The lesion depth of the conventional fluoride group D1 was around 70 μm, and the body of the lesion in the elevated fluoride group D2 was even smaller around 40 μm. The translucent zone did not differ in control groups and in the conventional fluoride group D1. However, this zone of demineralizing front was significantly more extended in the elevated fluoride group D2 representing mainly inhibition of demineralization in the inner part of the lesion in contrast to mineral uptake in the outer part of the human enamel lesion. There was also a sharp increase of laminations in close to half of all sections. In contrast, laminations were very rare in the remineralization group with no fluoride, and they were completely absent in the teeth with demineralization cycles only. In natural white-spot lesions laminations are attributed to short or long-lasting periods of different caries challenge [12,13]. Ten Cate et al. [2] showed that mineral uptake and loss occur at different depths within the lesions, and they assumed that fluoride-induced recrystallization has made the crystallites larger and less soluble in acid. Our PLM features confirm this hypothesis, and they demonstrate a clear dose response. The marked difference in the mineral structure of artificial incipient lesions, compared to that of sound enamel, gives rise to the phenomenon of simultaneous remineralization and demineralization. Quantitative microradiographic studies after application of higher fluoride concentration showed an increase in remineralization in the outer lesion and a decrease in demineralization in the inner part, resulting in a significant increase in mineral gain [3]. It has recently been discussed that with elevated external F^- ^levels, the F^- ^gradient might be higher, driving the fluoride deeper into the advanced lesion, in spite of the F^- ^diffusion being slowed by adsorption onto and reaction with hydroxyapatite crystallites [4]. According to these results higher concentrations of fluoride are required to prevent the progression of artificial caries-like lesions. Therefore, the F^- ^bioavailability in oral fluids simulated by the presented study design plays a significant role in enhancing remineralization of enamel caries lesions.

## Conclusion

In conclusion, remineralization and demineralization of advanced human enamel caries-like lesions were found to benefit from higher fluoride concentrations (4350 ppm versus 1450 ppm) during oral hygiene tablet treatment in a pH cycling model.

## Competing interests

The authors declare that they have no competing interests. Prodentum, Berlin, Germany provided the DETTABS^® ^for this investigation but had no further influence on the study design.

## Authors' contributions

PG was responsible for writing the manuscript. TK carried out the experiments and the measurements. WHA was responsible for the morphological studies.

All authors read and approved the final version of the manuscript.

## Pre-publication history

The pre-publication history for this paper can be accessed here:


